# Retroperitoneal Duodenal Diverticulum Microperforation: The Role of Computed Tomography

**DOI:** 10.7759/cureus.65494

**Published:** 2024-07-27

**Authors:** Dragan Vasin, Ksenija Mijovic, Aleksandar Pavlovic, Danica Stanic, Tijana Tomic

**Affiliations:** 1 Radiology Department, Faculty of Medicine, University of Belgrade, Belgrade, SRB; 2 Emergency Radiology Department, Center for Radiology, University Clinical Center of Serbia, Belgrade, SRB; 3 Emergency Surgery Department, University Clinical Center of Serbia, Belgrade, SRB

**Keywords:** upper gastrointestinal surgery, abdominal radiology, emergency medical service, computed tomography abdomen, duodenal diverticulum perforation

## Abstract

Duodenal diverticula are relatively common, but perforations are rare and therapy has not yet been standardized. The most common location of diverticula is the descending duodenum, usually on the lateral side next to the pancreas, so perforations present with an atypical clinical course. We present a case of a 73-year-old female patient with epigastric pain and nausea. Abdominal CT revealed an air-fluid collection near the laterocaudal border of the descending duodenum, suggestive of duodenal microperforation and incipient abscess formation. During the operation, a diverticulectomy was performed with primary duodenal closure and abdominal drainage. A diverticulum microperforation contains some extraduodenal air bubbles, no evidence of abscess, and free air in the subdiaphragmatic region. It is an imaging entity with distinct clinical and biochemical features, and radiological findings often determine the final decision on treatment.

## Introduction

Duodenal diverticula are relatively common, but perforations are rare and therapy has not yet been standardized. The most common location of diverticula is the descending duodenum, usually on the lateral side next to the pancreas, so perforations present with an atypical clinical course. The role of imaging is important in early detection so that intensive conservative treatment can be initiated promptly. Perforation of the duodenal diverticulum is exceedingly rare, with a total of 162 cases published in the world literature as of 2012 [1].

## Case presentation

**Figure 1:**
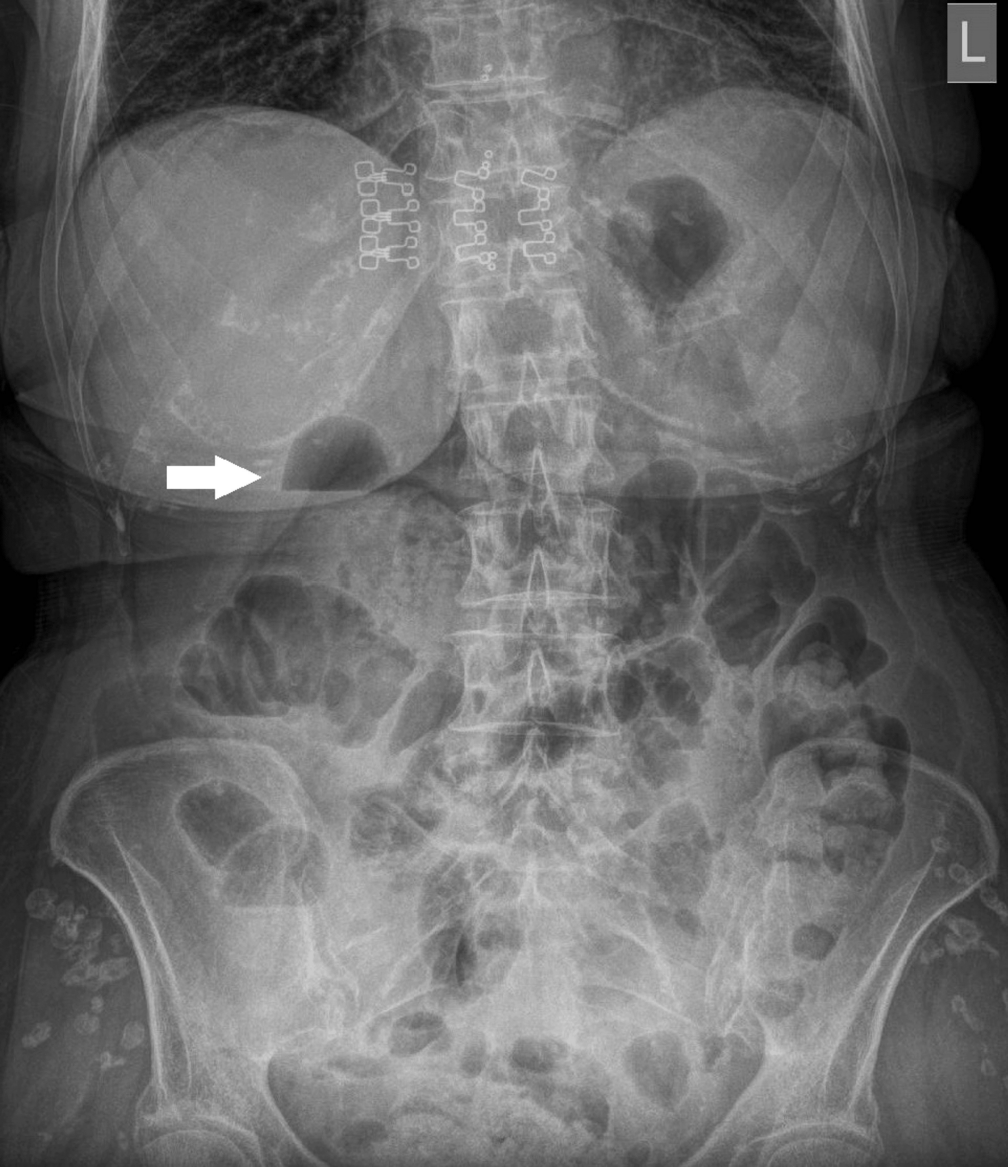
Plain abdominal radiography. A solitary gas-fluid level of the duodenum with luminal distention (white arrow) and several gas-fluid levels of the ascending colon and ileum in the right hemiabdomen, without signs of pneumoperitoneum or pneumoretroperitoneum.

A 73-year-old female patient without a previous history of ulcer disease presented to the emergency department with epigastric pain and nausea. Physical examination revealed right upper quadrant abdominal tenderness without signs of peritoneal irritation. Laboratory analyses showed leukocytosis (24 × 10^9^/L) and elevated C-reactive protein (243 mg/L). Plain abdominal radiography showed a solitary gas-fluid level in the duodenum with luminal distention, as well as several gas-fluid levels in the ascending colon and ileum in the right hemiabdomen, without signs of pneumoperitoneum or pneumoretroperitoneum (Figure 1).

**Figure 2:**
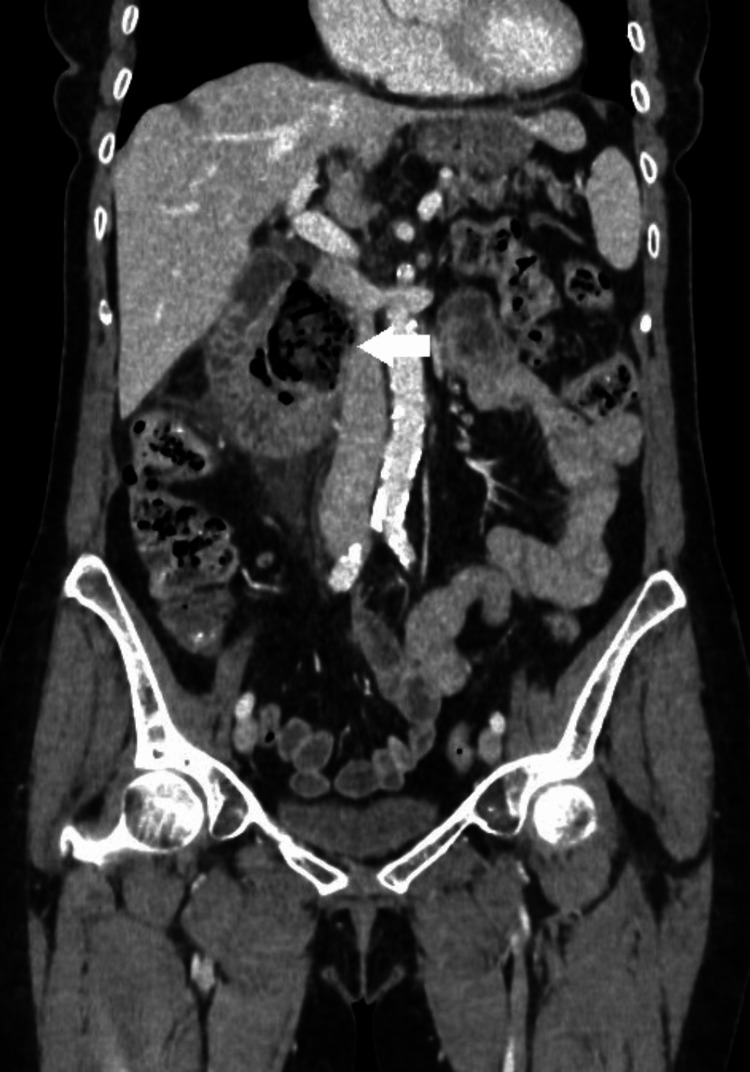
Initial CT of the abdomen and pelvis - coronal reconstruction. Duodenal diverticulum (white arrow) with duodenal wall edema, peridiverticular "fat-stranding," and extraluminal air bubbles - CT signs of perforation.

**Figure 3:**
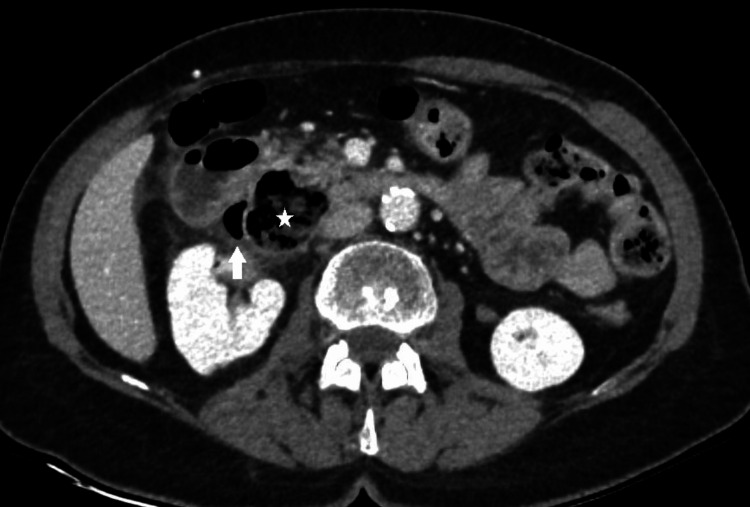
Initial CT of the abdomen and pelvis - axial image. Duodenal diverticulum (white star) localized on the medial wall of the descending duodenum at the pancreatic side with peridiverticular and periduodenal "fat-stranding" and a small amount of extraluminal gas (white arrow) near the medial duodenal wall - CT signs of perforation.

Ultrasound revealed a distended stomach and circumferential hypoechoic thickening of the duodenal wall with hyperechoic periduodenal fat tissue. A CT scan was performed with neutral peroral contrast because there was no suspicion of digestive tract perforation. Abdominopelvic CT showed uniform circumferential thickening of the descending duodenum wall with a 35 mm diverticulum at the pancreatic side of the descending duodenum with peridiverticular "fat-stranding" containing several free gas inclusions as indirect signs of perforation (Figures 2, 3).

There were no CT signs of peritonitis or free subphrenic air, leading to a diagnosis of retroperitoneal duodenal diverticulum perforation.

**Figure 4:**
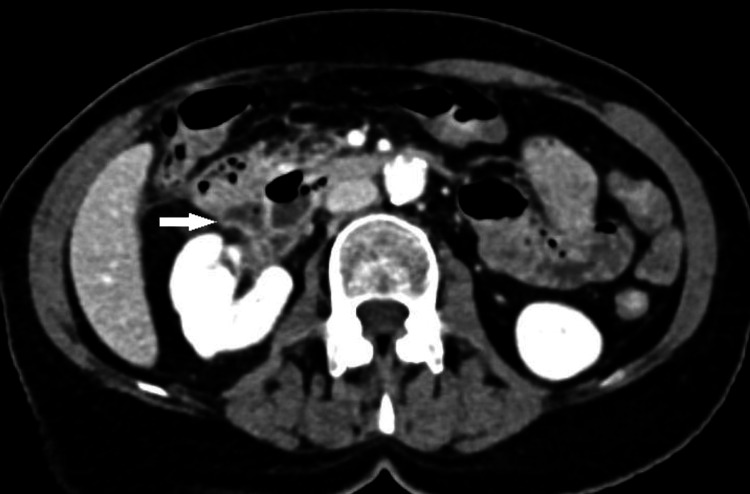
Follow-up CT of the abdomen and pelvis. Air-fluid collection near the posteromedial border of the descending duodenum with anterior pararenal fascia hypervascular thickening suggestive of incipient abscess formation (white arrow).

Upper endoscopy identified an inflamed duodenal diverticulum arising from the second portion of the duodenum 2 cm below the ampulla of Vater, without signs of perforation. Initially, conservative therapy was initiated and included hydration, bowel rest, intravenous proton-pump inhibitors, and broad-spectrum antibiotics but the patient’s white blood cell count continued to rise. Five days later, a repeat abdominal CT revealed a fluid collection near the posteromedial border of the descending duodenum with hypervascular thickening of the anterior pararenal fascia (Figure 4), suggestive of incipient abscess formation. Consequently, the patient underwent emergency surgery.

**Figure 5:**
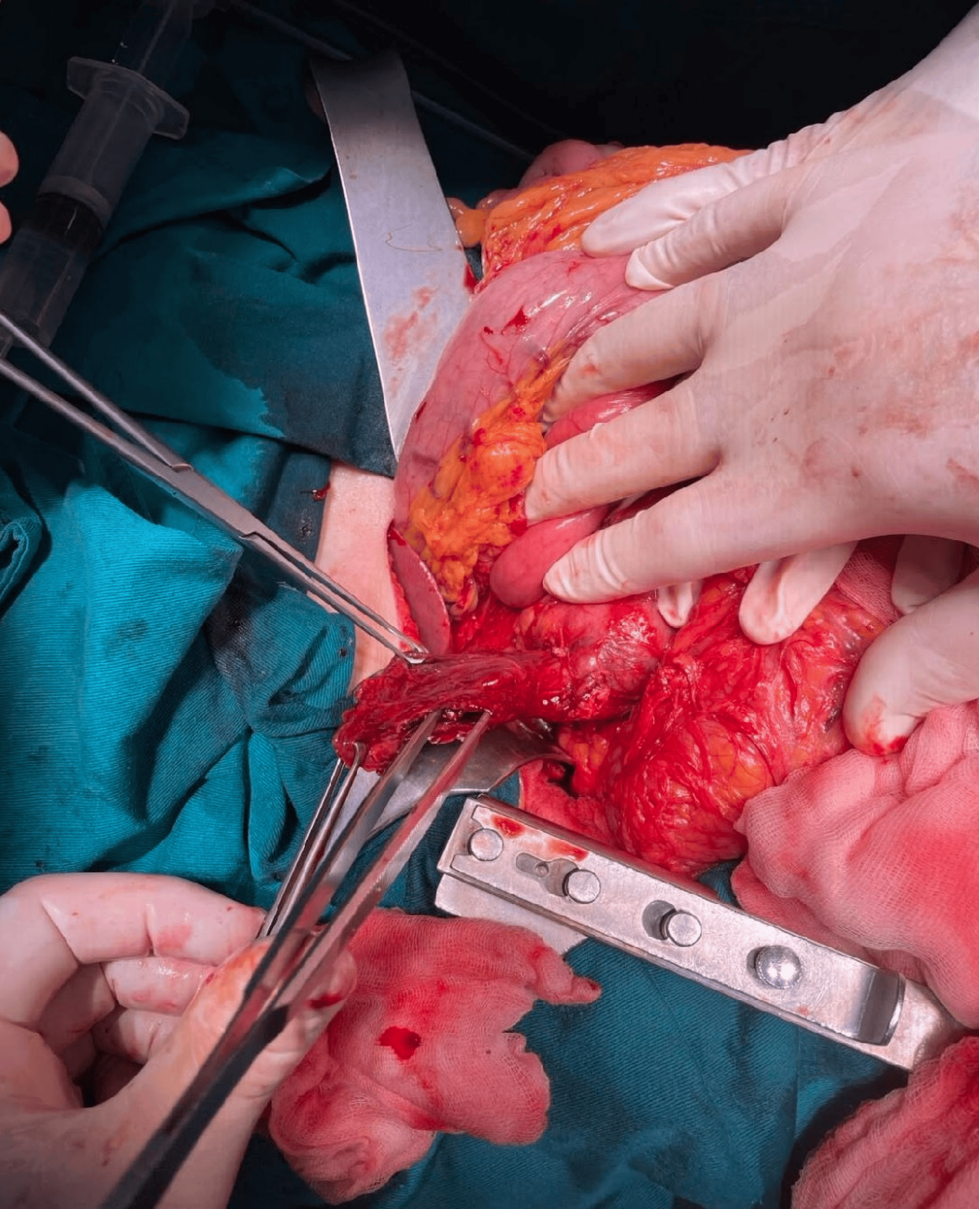
Intraoperative finding. Perforation site of the duodenal diverticula.

During surgery, an abscess was found due to perforation of a duodenal diverticulum, so a diverticulectomy was performed with primary duodenal closure and abdominal drainage (Figure 5).

**Figure 6:**
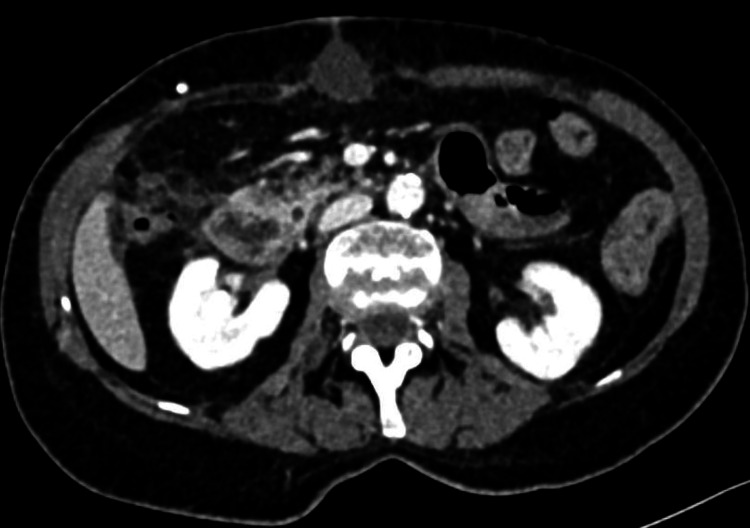
Postoperative CT of the abdomen and pelvis. Normal duodenal wall without periduodenal fluid collection.

The postoperative period was uneventful, the Levin tube was placed, and the patient started feeding on the fourth postoperative day. Postoperative CT was normal, and the patient was discharged home on day 10 after surgery (Figure 6).

## Discussion

The clinical course in a patient with a perforated duodenal diverticulum is often heterogeneous and non-specific [1]. Clinical imaging is an essential adjunct to our workup of a patient with acute symptoms and, in the majority of cases, will make a diagnosis or set the indication for operative treatment. Plain radiography and ultrasound do not offer much in the case of a perforated duodenal diverticulum, as free sub-diaphragmatic air appears in only about 10% of cases [2]. Key radiological findings on the initial CT exam are duodenal wall thickening ≥4 mm, mesenteric fat stranding, and extraluminal or retroperitoneal air/fluid [1]. Other criteria include the location and size of the duodenal diverticulum. Conservative treatment includes bowel rest, intravenous antibiotic therapy, parenteral nutrition, and combined endoscopic-percutaneous drainage of the retroperitoneal abscess. Close clinical and radiology observation is mandatory. In our case, follow-up CT showed increased retroperitoneal fluid with initial abscess formation, indicating the need for surgical intervention. There were no signs of peritonitis or free intraperitoneal air on either CT exam. Although duodenal diverticulum perforation may present similarly to duodenal perforation on CT, the number of preoperative diagnoses of duodenal diverticulum perforation has increased with the common use of CT [3]. Except for duodenal diverticula, other causes for duodenal perforation are iatrogenic, ulcer disease, or foreign body [4].

## Conclusions

The morphological diagnosis of retroperitoneal duodenal perforation is crucial for treatment planning, and frequent CT scans are necessary to detect complications such as retroperitoneal abscess development. Plain abdominal radiography and ultrasound are not sensitive radiological techniques due to the unfavorable anatomical position of the duodenum.

A diverticulum microperforation contains some extraduodenal air bubbles, no evidence of abscess, and free air in the subdiaphragmatic region. It is an imaging entity with distinct clinical and biochemical features, and radiological findings often determine the final decision on treatment.
